# Coping with the burden of the COVID-19 pandemic: a cross-sectional study of community pharmacists from Serbia

**DOI:** 10.1186/s12913-021-06327-1

**Published:** 2021-04-06

**Authors:** Jelena Jovičić-Bata, Nebojša Pavlović, Nataša Milošević, Neda Gavarić, Svetlana Goločorbin-Kon, Nemanja Todorović, Mladena Lalić-Popović

**Affiliations:** 1 University of Novi Sad, Faculty of Medicine, Department of Pharmacy, Hajduk Veljkova 3, Novi Sad, Serbia; 2 University of Novi Sad, Faculty of Medicine, Center for Medical-Pharmaceutical Research and Quality Control, Hajduk Veljkova 3, Novi Sad, Serbia

**Keywords:** Community pharmacy, Community pharmacist, Work environment, Job-related stress, Coronavirus, COVID-19

## Abstract

**Background:**

Rapid spread of COVID-19 forced the public to turn to community pharmacies as the most accessible points of primary healthcare, overloading pharmacy services. The objectives of this research were to detect and describe the changes in work environment of community pharmacists in Vojvodina during the state of emergency due to COVID-19 pandemic. Moreover, the COVID-19 pandemic effects on job related stress were assessed.

**Methods:**

Community pharmacists from Vojvodina completed an online questionnaire on work environment changes related to COVID-19 (cross-sectional study).

**Results:**

Out of the 1574 licenced pharmacists in Vojvodina, 392 completed the survey. Workload increase, reported by 90.8% of pharmacists, was caused mostly by higher demand for safety equipment, antiseptics and disinfectants, dietary products and medicines. Most pharmacists (93.1%) considered pharmacy workflow to be more complex than before the pandemic. Clients’ behavior was described as less pleasant since the start of the pandemic by 67.6% of the community pharmacists. Many were concerned for their health and the health of their families (68.9%). Community pharmacists rated their stress levels higher if they i) were working in larger chains, ii) experienced clients’ behavior as less pleasant or/and iii) were concerned for their/their family health.

**Conclusions:**

Current research pointed out the need for a more robust healthcare system which would allow rapid introduction of new activities and roles for community pharmacists that could possibly decrease job-related stress. Legal steps to improve the work environment in community pharmacies are necessary and urgent in order to fully utilize their skills and knowledge.

**Supplementary Information:**

The online version contains supplementary material available at 10.1186/s12913-021-06327-1.

## Introduction

Severe acute respiratory syndrome caused by coronavirus 2 (SARS-CoV-2) was first identified in December 2019 in China. The first case of COVID-19 in Serbia was reported on March 6, 2020. On March 11, 2020, the World Healthcare Organization (WHO) declared coronavirus disease (COVID-19) outbreak a global pandemic [1]. WHO called upon the swift action of governments and societies to prepare themselves for the pandemic, bettering their emergency response systems, communicating the risks and educating the public on means of protection. This decision has societal, political and economic consequences that are hard to fully foresee at this moment, but its effect on healthcare systems is already palpable. The pandemic has strained health systems worldwide to their limits [2–4] causing the public to turn to community pharmacists as the most accessible primary healthcare providers. Community pharmacies are frequently the first point of contact with the healthcare system with enormous potential to relieve the burden of COVID-19 pandemics for other healthcare providers, allowing them to focus on more severe cases [5]. During these challenging times, the importance of pharmacy personnel as front-line healthcare workers and their potential is starting to be fully recognized by both, the public and the healthcare system. The ongoing transformation of pharmacy from medicine-centered to patient-centered care [6] might be accelerated by the pandemic, but the effects of such rapid changes on pharmaceutical workforce are unclear [7, 8]. Continual threat of infection together with changes in supply chains, workflow and work routines, difficult clients’ behavior, managing crowding and social distancing in pharmacies have been shown to be significant stressors [7, 9] with potential to increase work-related stress [7, 10] of pharmacy teams.

In Serbia, state of emergency that was in effect from March 15 to May 6, 2020, affected many community pharmacies’ activities, from procurement to provision of pharmaceutical care, due to limited movement and imposed curfew. At the moment of submission of this article, there were 271,364 of registered cases of COVID-19 and 2380 death cases in Serbia (mortality ratio 0.88%), according to the official data [11]. However, data on the effect of pandemic on community pharmacists in Serbia are not available.

The aims of this study were to detect and describe the changes in work environment of community pharmacists in the Autonomous Province of Vojvodina^1^ during the state of emergency due to COVID-19 pandemic and to assess the effect of those changes on job-related stress.

## Methods

This research was an observational, cross-sectional study, carried out in a sample of 392 pharmacists working in community pharmacies in Vojvodina in April and May 2020, during the state of emergency due to COVID-19 pandemic.

### Research instrument

A 36-items questionnaire was developed to assess the changes in work environment of community pharmacists during the COVID-19 pandemic (Additional file 1). Face and content validity of the questionnaire were reviewed by a group of 5 pharmacists: 3 community pharmacists (30, 41 and 65 years old) and 2 academic pharmacists with previous experience in community pharmacies of 5+ years (minimizing questionnaire bias). In response to reviewers’ suggestions, one question was moved to a different section of the questionnaire. Questionnaire was administered online using Google Forms. Online form was tested for practical issues by a group of 9 community pharmacists (7 females, age range 26–61 y), but their feedback did not lead to any changes being made.

The invitation text provided information on study rationale and goals, the concept of anonymity and voluntary nature of participation in the study. A short explanation on data handling and management was given. Research team was introduced and contacts were provided in case of any issues or questions. Potential participants were then asked to confirm they are: 1) pharmacists currently working in community pharmacies in Vojvodina and 2) willing to participate in the study (eligibility criteria). It was clearly stated that answering “yes” was considered as a consent for their participation. Upon confirmation, the participants answered questions on general information, workload, workflow, interactions with clients, work conditions and personal perceptions of different aspects of their job. Skip logic was used to ensure participants get to answer only questions that are relevant to them according to their previous answers.

### Sample

According to the data of the Pharmaceutical Chamber of Serbia (PCS), there were 1574 registered pharmacists in Vojvodina licenced for work in community pharmacies at the moment of the study (study population). Using power analysis, the minimum sample size for the said population was determined to be 309 (confidence level 95% (α = 0.05), margin of error 5%).

An invitation e-mail was sent by a representative of the PCS to all 1574 registered community pharmacists from Vojvodina (using the PCS register) in compliance with the current national Law on the Protection of Personal Data. Over a period of 3 weeks, 392 community pharmacists completed the survey (voluntary response sampling, response rate 24.9%).

### Statistics

Statistical analysis was performed using SPSS Statistics for Windows, ver. 24 (IBM Corporation).

Categorical variables were characterized using frequencies, while means and standard deviations were used to describe continuous variables. Where appropriate, numerical variables were transformed into categorical for the purpose of interpretation. χ2 test was used to assess differences in categorical variables between groups, followed by post hoc testing using the proportion test. Ordinal regression analysis was used to assess the dependence of the perception of job-related stress (3-point Likert scale; high, moderate, low) on a set of 10 predictor variables (categorical and ordinal).

A *p* value of < 0.05 was considered statistically significant.

## Results

Out of the 1574 eligible pharmacists, 392 community pharmacists were included in the sample. Most of the participants were females, working in community pharmacies that are part of large pharmacy chains and in urban areas (Table 1). High proportion of female pharmacists in the sample corresponds to the proportion of females in the study population (91.1%).

**Table 1:** General characteristics of community pharmacists in the sample (*N* = 392)

	N/Mean	%/SD
Gender		
Male	30	7.7
Female	362	92.3
Age, y	36.2	9.7
< 35	207	52.8
35–44	117	29.8
45+	68	17.3
Experience in community pharmacy, y	9.6	9.0
< 10	244	62.2
10+	148	37.8
Job position		
Responsible pharmacist	227	57.9
Pharmacist	165	42.1
Type of pharmacy		
Chain of ≤4 pharmacies	29	7.4
Chain of 5–15 pharmacies	75	19.1
Chain of >15 pharmacies	265	67.6
Independently owned	23	5.9
Pharmacy location		
Urban area	300	76.5
Suburban area	59	15.1
Rural area	33	8.4

Percentages may not add up to 100.0 due to rounding

### Workload

Most respondents reported an increase in their workload during the COVID-19 pandemic (90.8%) as shown in Table 2. Differences were observed in the proportion of pharmacists reporting the changes in their workload by gender (*p* = 0.032), age (*p* = 0.003), years of experience (*p* = 0.031) and type of pharmacy (*p* = 0.002). Increase in workload was more often reported by women (*p* = 0.033), by younger pharmacists (<35y) in comparison to 45+ (*p* < 0.001), respondents with < 10 years of experience (*p* = 0.021) and those working in chains of 15+ pharmacies compared to independently owned (*p* < 0.001) (see Additional file 2).

**Table 2:** Perception of workload, workflow, interactions with clients and availability of protection equipment during COVID-19 pandemic among community pharmacists in Vojvodina

	N	%
Workload		
Increase	356	90.8
No change	23	5.9
Decreased	13	3.3
Workflow		
Changed	365	93.1
Not changed	27	6.9
Interactions with clients		
More pleasant	53	13.5
Unchanged	74	18.9
Less pleasant	265	67.6
Availability of protection equipment		
Not always available	204	52.0
Always available	188	48.0

The dominant reasons for the increased workload were (*N* = 356) higher demand for safety equipment (e.g. gloves, masks) (97.8%), antiseptics and disinfectants (96.9%), dietary products (83.7%) and medicines (62.9%), followed by disinfecting procedures, higher prescription load, counseling and compounding.

Notable increase in demand for analgesics and antipyretics was observed by 94.1% of community pharmacists, for antiseptics by 84.6% pharmacists, anxiolytics, sedatives and hypnotics (49.7%), antimalarials (44.1%), antivirotics (21.9%), antihypertensives (18.8%) and extemporaneous medicines (17.7%) and antibiotics, namely azithromycin. Stocking up on medications for chronic conditions by clients was also reported.

Dietary products/supplements reportedly in high demand were vitamin C (noted by 97.8% of the respondents), zinc (97.8%), multivitamin-multimineral supplements (71.9%), beta-glucans (57.9%), germanium and ganoderma (46.1%), bee product-based supplements (45.8%), as well as echinacea, alkylglycerols, valerian, melatonin, aloa, noni, vitamin D and selenium-containing antioxidant supplements.

Shortages of safety equipment were reported by 98.0% of community pharmacists, of antiseptics and disinfectants by 96.6%, dietary products and medicines by 59.6 and 58.7%, respectively. To overcome the shortages, surveyed community pharmacists reported setting/limiting the number of product units per purchase for clients (85.4%), communicating with suppliers more often (68.3%) and compounding of solutions and gels in 36.5% of the cases.

### Workflow

Majority of community pharmacists (93.1%) considered pharmacy workflow to be affected by the COVID-19 pandemic (Table 2). Reporting workflow changes differed by gender (*p* = 0.028) and job position (*p* = 0.023). Females claimed that workflow was affected more often than males (*p* = 0.023), as well as responsible pharmacists when compared to pharmacist in non-managing positions (*p* = 0.028). New processes were interpreted as more complex than before the pandemic by 61.4% of pharmacists (see Additional file 3).

### Interactions with clients

Community pharmacists rated clients’ demeanor during the COVID-19 pandemic mostly as less pleasant than before (67.6%) (Table 2). Those from the < 35 years-group rated clients’ behavior as less pleasant more often than older pharmacists (35–44 and 45+ y) (*p* = 0.011, *p* < 0.001, respectively). Respondents with 10+ years of experience observed more pleasant clients’ behavior than pharmacists with < 10 y experience (*p* = 0.005). Pharmacists working in chains of ≤4 pharmacies rated their clients’ demeanor as pleasant more frequently than those in chains with 5–15 and >15 pharmacies (*p* = 0.030 and *p* < 0.001, respectively) (see Additional file 4). One third of surveyed pharmacists (33.7%) noted that clients did not follow the recommended safety measures.

### Working conditions

Safety equipment was always available to 48.0% of the participants, while the other 52.0% had experienced occasional shortages (see Table 2 and Additional file 5). Protective equipment was provided completely or partially by the employer to 53.8 and 42.3% of pharmacists, respectively.

One percent of the respondents did not use any safety equipment. Protective masks and gloves were most commonly used (by 95.9 and 95.2% of pharmacists, respectively). Physical barriers were available in pharmacies of 75.5% of respondents.

### Job-related personal perceptions of pharmacists

Majority of the respondents were concerned for their/their families’ health (68.9%) during the pandemic. Inaccurate and incomplete reporting about COVID-19 related issues in the media affected the work of 75.0% of surveyed pharmacists. Many noticed the lack of time to devote to patients (72.7%). Most (95.4%) considered physical barriers between pharmacy employees and clients to be necessary.

The possibility of compounding during the pandemic was perceived as an activity that could improve pharmaceutical care by 74.4% of pharmacists. Pharmacists believed they should be authorized to independently renew prescriptions for stable chronic conditions in 46.2% of the cases, while 40.8% were opposed to the idea. The potential role of pharmacists in immunization against COVID-19 was supported by 29.6% of respondents.

### Regression model

Ordinal regression model was used to assess the effect of community pharmacists’ characteristics and changes in work environment on job-related stress perception. The model was statistically significant (χ^2^ = 77.669, *p* < 0.001) and explained 24.1% of the results variance (Table 3). Community pharmacists working in chains of 5–15 and >15 pharmacies were 3.088 and 2.795 times more likely to rate their job-related stress higher (*p* = 0.042 and *p* = 0.043, respectively) in comparison to pharmacist working in independently owned pharmacies. Pharmacists reporting their clients behavior as less pleasant were 2.079 more likely to rate their job-related stress higher (*p* = 0.029). Pharmacists expressing low concern for their/their families’ health were less likely to report high levels of job-related stress during the pandemic (OR = 0.157, *p* < 0.001).

**Table 3:** Ordinal regression model: Odds ratios (OR) and 95% confidence intervals (CI) for the association of community pharmacists’ characteristics and changes in work environment with job-related stress perception

	OR	CI 95%	*p*
Experience in community pharmacy, y	1.017	0.985–1.050	0.306
Gender			
Male	0.814	0.331–2.005	0.655
Female	1^a^		
Job position			
Responsible pharmacist	1.106	0.646–1.895	0.713
Pharmacist	1^a^		
Pharmacy			
Chain of ≤4 pharmacies	2.134	0.599–7.603	0.242
Chain of 5–15 pharmacies	3.088	1.042–9.15	0.042
Chain of >15 pharmacies	2.795	1.033–7.561	0.043
Independently owned	1^a^		
Pharmacy location			
Urban area	0.635	0.236–1.711	0.369
Suburban area	0.836	0.258–2.708	0.765
Rural area	1^a^		
Workload			
Decreased	0.574	0.158–2.087	0.400
Unchanged	1.333	0.421–4.220	0.625
Increased	1^a^		
Workflow			
Unchanged	1.211	0.432–3.396	0.716
Changed	1^a^		
Interactions with clients			
More pleasant	0.691	0.302–1.579	0.381
Less pleasant	2.079	1.076–4.017	0.029
Unchanged	1^a^		
Availability of protective equipment			
Always available	1.097	0.652–1.846	0.727
Not always available	1^a^		
Concern for own health or the health of their families			
Low	0.157	0.089–0.277	< 0.001
Neither low or high	0.435	0.188–1.004	0.051
High	1^a^		

^a^reference category

## Discussion

Our study detected significant changes in work environment of community pharmacists in Vojvodina at the times of COVID-19 pandemic.

### Workload

During the COVID-19 pandemic, community pharmacists’ workload has drastically increased worldwide [9, 12–15]. Our respondents’ workload increased mainly by the surge in demand for specific pharmaceutical products. Increase in workload has affected females, younger, less experienced and pharmacists in larger chains more severely. Females’ perception might have been intensified by the higher burden of domestic work, unpaid care for children and older family members during the pandemic [16]. Many pharmacies engaged older employees in administrative tasks, perceived as more safe and resilient [9]. Such reorganization left the younger and the less experienced pharmacists at the counter, dealing with clients, possibly causing them to notice the increase in workload more. Larger workload in bigger chains was previously observed by researchers [17, 18].

Information on COVID-19 infection management presented by the health authorities, the researchers and the media [19–21] caused high demand for specific medicines. The observed surge in demand for anxiolytics, sedatives and hypnotics implies the possible negative effect of the pandemic (and/or mechanisms to fight it) on mental health of our population – an indirect effect of COVID-19 [22].

Dietary supplements with claimed immunomodulatory or antiviral effect were the most sought-after, similar to other countries [23]. Bee product-based supplements were in high demand, as they are traditionally highly valued and more often used [24] in the region.

Shortages of safety equipment, disinfectants, antiseptics, medicines and dietary products are a global problem [25–28]. All of our subjects have noticed shortages of the aforementioned products. Clients’ stocking up on chronic medication, possibly out of fear and uncertainty [13, 15, 29], present an added strain on the healthcare system. In attempts to overcome shortages, community pharmacists in Vojvodina resorted most often to limiting the number of products per purchase, as suggested by WHO [30] and sourcing from multiple suppliers, as pharmacists in other countries [21]. Only one third of surveyed pharmacists started compounding solutions and gels, although it has been recommended by WHO [30], possibly due to shortage of alcohol and other disinfecting substances and compounding-related legal limitations. Several European countries made legal changes to allow for compounding at pharmacies with no registered compounding services to mitigate the shortages [31]. Reluctance of pharmacists to start compounding might be linked to the lack of knowledge, confidence or instructions for preparation [29]. As compounding is a specific pharmacists’ skill, community pharmacists should be encouraged to compound deficient products and adopt more proactive attitudes towards compounding [29].

### Workflow

Changes in workflow noticed by surveyed pharmacists were mostly administrative and procurement-related. Although changes were introduced to simplify pharmacists’ work during the pandemic, most pharmacists found them more complex and many shared negative thoughts and experiences with the new processes. Job-related changes are associated with feelings of anxiety, resistance [32], insecurity, loss of control and general inconvenience [33]. Employee training and education could be helpful strategies in increasing employees’ adaptability [32], but during the pandemic, these were not possible.

### Interactions with clients

The usual pharmacist-client interactions were strained during the pandemic due to clients’ amplified emotional responses – fear, anxiety, panic-buying and/or non-conforming to preventive measures [9, 15, 34], as reported by two thirds of surveyed pharmacists. Younger pharmacists rated clients’ behavior as less pleasant more frequently, possibly because older pharmacist had limited interactions with clients. Pharmacists from bigger organizations reported clients’ behavior being less pleasant during the pandemic in comparison to those from smaller chains. This could stem from higher general satisfaction of clients/patients with independent pharmacies in comparison to chain pharmacies [35].

### Working conditions

Recommendations on the use of personal protective equipment (PPE) varied considerably across countries [27]. Almost all of our respondents used masks and gloves, although national recommendations on general protective measures in community pharmacies were published after the data acquisition for this research has been finished (requiring distancing and mask use at all times) [36]. Globally, due to supply problems, personal protective equipment was not always available to pharmacists [34, 37, 38]. In our study, community pharmacists experienced occasional shortages of safety equipment.

### Job-related personal perceptions of pharmacists

Pharmacists across the world were highly concerned for their/their families’ health [34, 39–41], similar to pharmacists enrolled in our study. Many surveyed pharmacists reported their work to be negatively affected by misinformation on COVID-19 circulated by the media. In an unprecedented “infodemic”, inaccurate and incomplete information about coronavirus spread rapidly adding to the fear and the anxiety of the public [39, 42, 43]. Most of respondents reported not having enough time to devote to clients, but such lack of time was observed even in non-pandemic circumstances [44].

Worldwide PPE shortages pressured many pharmacies to install physical barriers in order to reduce the risk of coronavirus infection of pharmacy teams and clients [15]. Majority of the respondents believed such barriers are necessary. After community pharmacists’ plea, PCS appealed to the National Ministry of Health to make physical barriers mandatory [45].

Dispensing of repeat prescriptions by community pharmacists could relieve some of the burden off the other healthcare workers [13]. Due to pandemic, some countries have already allowed community pharmacists to renew chronic treatment prescriptions [31]. In Serbia, chronic treatment prescription (eTherapy) validity period was extended from 6 to 9 months. Patients had to request the extension from their GPs, leaving the expertise of community pharmacists underutilized. Half of our respondents believed community pharmacists should be authorized to extend eTherapy during the pandemic without consulting patients’ GP. Many opposed that idea, suggesting they do not recognize their potential in chronic patients’ care.

Community pharmacists present a great potential in increasing the number of administered doses of vaccines and reducing the time to achieve high coverage [46]. Active participation of pharmacists in vaccination is not foreseen by relevant legislation in Serbia, which might explain the low percentage of pharmacist with positive attitudes towards active participation in vaccination.

### Regression model

Community pharmacists in Vojvodina rated their job-related stress higher if they worked in larger pharmacy chains, if they believed their clients behavior was less pleasant than before the pandemic and if they were more concerned for their/their families’ health. Pharmacists working in larger chains have been shown to be under more stress, mainly due to high prescription volumes, time constraints, overtime etc. [47]. Clients’ inappropriate behavior and concern for their own/family members’ health has also been shown to cause stress and reduce resiliency in pharmacy settings [9, 48–50]. In addition, our research was carried out at the beginning of the pandemic, while protective equipment supply was unstable and vaccines were not available, which might explain the contribution of fear of contracting the coronavirus and infecting family members to pharmacists’ stress levels.

### Study limitations and strengths

Main advantage of the presented research is a unique perspective on the changes in work environment of community pharmacies in the state of emergency during COVID-19 pandemic. Direct feedback from surveyed pharmacists provided invaluable insight into genuine thoughts regarding the pharmaceutical care provided by community pharmacists during the pandemic.

Due to rapidly changing circumstances, our research used a non-standardized questionnaire. Our results have limited generalizability, due to sampling method, as well as specific regional demographic and social characteristics. All limitations of the cross-sectional study design, as well as positive and negative aspects of online surveying apply to our study (more honest answers, fast, cheap, easy to use for participants and researchers vs. lower response rates, non-response bias).

To the best of authors’ knowledge, this is the first study of this kind conducted in Serbia.

## Conclusion

The global COVID-19 pandemic increased the workload and altered the workflow of community pharmacists in Vojvodina. Pharmacists working in larger chains, reporting clients’ behavior as less pleasant and those who were more concerned for their/their families’ health during the pandemic reported higher levels of job-related stress.

Presented findings highlight the opportunity for pharmacists to be recognized as healthcare providers on the frontline of the healthcare system. Current research pointed out the need for more robust healthcare system which would allow rapid introduction of new activities/roles for community pharmacists in order to fully utilize their skills and knowledge. Legal steps to improve the work environment in community pharmacies and better definition of community pharmacists’ role in the healthcare system are necessary and urgent.

## Supplementary Information

**Additional file 1.**

**Additional file 2.**

**Additional file 3.**

**Additional file 4.**

**Additional file 5.**

## Data Availability

Data are available from the corresponding author on a reasonable request.

## Abbreviations

WHO: World Health Organization
COVID-19: Coronavirus disease
PCS: Pharmaceutical Chamber of Serbia
SPSS: Statistical Package for Social Sciences

## Footnotes

1 The Autonomous Province of Vojvodina is located in the north of Serbia with the population of close to 2 million according to the 2011 Census (28% of the population of Serbia) and covering 25% of the area of Serbia.
