# Supplementary material for: Coping with the burden of the COVID-19 pandemic: a cross-sectional study of community pharmacists from Serbia
Source: BMC Health Serv Res. 2021 Apr 6;21:304. doi: 10.1186/s12913-021-06327-1 (PMC8022120; doi:10.1186/s12913-021-06327-1)
Supplement: Supplementary file 1 — Additional file 1. [file 12913_2021_6327_MOESM1_ESM.docx]

Additional file 1. English translation of the questionnaire (translated from Serbian)

ONLINE SURVEY

**Changes in work environment of community pharmacists during the COVID-19 pandemic**


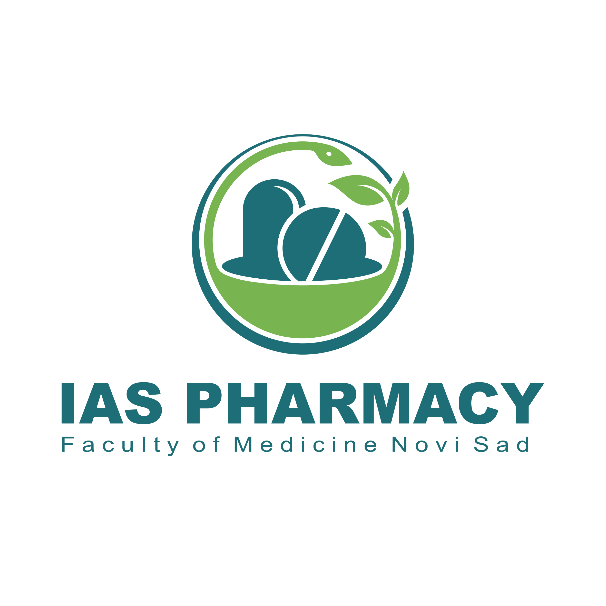


**Department of Pharmacy**

Faculty of Medicine, University of Novi Sad

April 2020

**General characteristics**

The following questions are about You and the community pharmacy You work at.

1. Gender
   1. male
   2. female
2. Age: ____________ (fill in the number)
3. Years of work experience in community pharmacy: ______________ (fill in the number)
4. Your position in the community pharmacy You work at could be best described as:
   1. responsible/chief pharmacist
   2. pharmacist
5. Community pharmacy You work at is:
   1. part of a chain of community pharmacies with ≤ 4 pharmacies
   2. part of a chain of community pharmacies with 5 to 15 pharmacies
   3. part of a chain of community pharmacies with > 15 pharmacies
   4. independent
6. Community pharmacy You work at pharmacies has an agreement with the National Health Insurance Fund.
   1. yes
   2. no
7. Community pharmacy You work at is located in:
   1. urban area
   2. suburban area
   3. rural area

**Workload**

The following questions are about Your workload during the COVID-19 pandemic.

1. Since the beginning of COVID-19 pandemic, workload in the pharmacy You work at:

*(participants choosing the answer 8.1 or 8.2 were automatically tranfered to question 17) ^[[1]](#footnote-1)^*

- 1. decreased
  2. did not change
  3. increased

1. In Your opinion, increase in workload is caused by:

*(participants were informed they are free to choose more than one question)*

- 1. more prescriptions
  2. increase in demand for antiseptics and disinfectants
  3. increase in demand for protective equipment (masks, gloves)
  4. increase in demand for certain groups of medications
  5. increase in demand for dietary supplements
  6. increase in demand for compounding of antiseptics and disinfectants
  7. increase in counseling activities on COVID-19-related issues
  8. more complex disinfecting procedures
  9. other reasons

1. If Your answer to the previous question was “other reasons”, please cite those reasons.

*(answer not required)*

_______________________________________________

1. Demand increased for the following medication groups:

*(participants were informed they are free to choose more than one question)*

- 1. analgesics and antipyretics
  2. antivirotics
  3. antimalarials
  4. antihypertensives
  5. anxiolytics, sedatives and hypnotics
  6. antiseptics
  7. compounded medicines
  8. galenic preparations
  9. other
  10. demand for medicines did not change

1. If Your answer to the previous question was “other”, plese note the specific groups of medicines.

*(answer not required)*

_______________________________________________

1. Demand increased for the following dietary supplements

*(participants were informed they are free to choose more than one question)*

- 1. vitamin C
  2. zinc
  3. multivitamins/multiminerals
  4. ehinacea
  5. beta glucan
  6. germanium/ganoderma
  7. bee products - propolis, royal jelly
  8. alkylglycerols
  9. aloa, noni
  10. valerian, melatonin
  11. other
  12. demand for dietary supplements did not change

1. If Your answer to the previous question was “other”, plese note the specific dietary supplements.

*(answer not required)*

_______________________________________________

1. Since the start of the pandemic, shortages happened of which of these items:

*(participants were informed they are free to choose more than one question)*

- 1. antieptics and disinfectants
  2. safety equipment (masks, gloves)
  3. certain groups of medicines
  4. certain dietary supplements
  5. there were no shortages

1. What steps did the pharmacy You work at undertook to overcome shortages?

*(participants were informed they are free to choose more than one question)*

- 1. compounding (solutions, gels)
  2. frequent communication with suppliers
  3. limiting the number of product units per purchase
  4. no specific steps were undertaken
  5. there were no shortages

**Workflow**

The following questions are about the workflow during the COVID-19 pandemic.

1. Since the beginning of COVID-19 pandemic, workflow in the pharmacy You work at:

*(participants choosing the answer 17.1 were automatically tranfered to question 21)*

- 1. did not change
  2. did change

1. New procedures intended to lower the risks of COVID-19 transmission were implemented at the pharmacy You work at
   1. yes
   2. no
2. New procurement-related procedures are
   1. more simple
   2. more cmplex
   3. these proceSdures did not change
3. What aspects of procurement-related procedures do You find more simple/more complex than before.

*(answer not required)*

_______________________________________________

**Interactions with clients**

The following questions are about the interactions with clients during the COVID-19 pandemic in the pharmacy You work in.

1. Since the start of the COVID-19 pandemic, clients behavior in the pharmacy You work in:
   1. did not change
   2. is more pleasant than before the pandemic
   3. is less pleasant than before the pandemic
2. Since the start of the COVID-19 pandemic, clients in the pharmacy You work in:
   1. are calm and respecting towards the recommended preventive measures
   2. did not respect the recommended preventive measures
3. Since the start of the COVID-19 pandemic, clients are asking for advice or counseling:

*(participants choosing the answer 23.2 or 23.3 were automatically tranfered to question 25)*

- 1. more often than before
  2. less than before
  3. there is no change in the frequency of clients asking for advice of counselilng

1. Since the start of the COVID-19 pandemic, clients are more often interested in advice or counseling on:

*(participants were informed they are free to choose more than one question)*

- 1. rational use of medicines
  2. rational use and/or at-home preparation of antiseptics and disinfectants
  3. nutrition
  4. rational supplementation
  5. disease prevention
  6. other

1. When advising or counseling about COVID-19, You rely mostly on guidelines provided by:
   1. World Health Organization
   2. Ministry of Health of the Republic of Serbia
   3. Pharmaceutical Chamber of Serbia
   4. other

**Work conditions**

The following questions are about the work conditions in the pharmacy You work in during the COVID-19 pandemic.

1. What safety equipment do You use while working with clients?

*(participants were informed they are free to choose more than one question)*

- 1. mask
  2. gloves
  3. protective gown
  4. face shield
  5. glass (or similar material) barrier to clients
  6. disinfecting barrier at the entrance to the pharmacy (shoe sanitizer mat)
  7. UV lamp
  8. other
  9. I do not use any safety equipment

1. Since the start of the COVID-19 pandemic, safety equipment in the pharmacy You work in were::
   1. always available in sufficient amounts/quantities
   2. not always available in sufficient amounts/quantities
2. Safety equipment during COVID-19 pandemic was:
   1. provided by Your employee
   2. provided by yourself
   3. partially provided by Your employee, partially provided by yourself
   4. not provided

**Personal attitudes**

The following questions are about Your personal attitudes towards COVID-19 related issues.

1. Please rate Your level of concer for Your/Your family’s health during the COVID-19 pandemic.
   1. High
   2. Low
   3. Neither low, nor high
2. Because of the COVID-19 pandemic, I don’t have enough time to devote to clients.
   1. I agree
   2. I disagree
   3. Neither agree, nor disagree
3. Inaccurate and incomplete reporting about COVID-19 related issues in the media impeded my work.
   1. I agree
   2. I disagree
   3. Neither agree, nor disagree
4. Physical barrier (glass or similar material) to clients in pharmacies are necessary.
   1. I agree
   2. I disagree
   3. Neither agree, nor disagree
5. Compounding could possibly improve pharmaceutical care during the COVID-19 pandemic.
   1. I agree
   2. I disagree
   3. Neither agree, nor disagree
6. Pharmacists should be authorized to independently renew prescriptions for stable chronic conditions during the COVID-19 pandemic.
   1. I agree
   2. I disagree
   3. Neither agree, nor disagree
7. Pharmacists should be authorized (after additional training) to participate in the COVID-19 immunization process.
   1. I agree
   2. I disagree
   3. Neither agree, nor disagree
8. How would you rate you job-related stress level during the COVID-19 pandemic?
   1. high
   2. moderate
   3. low

1. NOTE: Italic font was used to highlight functions that were available in online form, but are not available in pdf documents [↑](#footnote-ref-1)
