# Supplementary material for: Coping with the burden of the COVID-19 pandemic: a cross-sectional study of community pharmacists from Serbia
Source: BMC Health Serv Res. 2021 Apr 6;21:304. doi: 10.1186/s12913-021-06327-1 (PMC8022120; doi:10.1186/s12913-021-06327-1)
Supplement: Supplementary file 2 — Additional file 2. [file 12913_2021_6327_MOESM2_ESM.docx]

Additional file 2.

Changes in workload of community pharmacists in Vojvodina during the COVID-19 pandemic by groups.

|  | | Workload during COVID-19 pandemic | | | | | | p |
| --- | --- | --- | --- | --- | --- | --- | --- | --- |
|  | | Decreased | | No change | | Increase | |  |
|  | | N | % | N | % | N | % |  |
| Total | | 13 | 3.3 | 23 | 5.9 | 356 | 90.8 |  |
| Gender | male | 1 | 7.7 | 5 | 21.7 | 24 | 6.7 | 0.032 |
|  | female | 12 | 92.3 | 18 | 78.3 | 332 | 93.3 |  |
| Age, y | <35 | 2 | 15.4 | 9 | 39.1 | 196 | 55.1 | 0.003 |
|  | 35-44 | 5 | 38.5 | 6 | 26.1 | 106 | 29.8 |  |
|  | 45+ | 6 | 46.2 | 8 | 34.8 | 54 | 15.2 |  |
| Experience, y | <10 | 4 | 30.8 | 12 | 52.2 | 228 | 64.0 | 0.031 |
|  | 10+ | 9 | 69.2 | 11 | 47.8 | 128 | 36.0 |  |
| Job position | responsible pharmacist | 8 | 61.5 | 16 | 69.6 | 203 | 57.0 | 0.480 |
|  | pharmacist | 5 | 38.5 | 7 | 30.4 | 153 | 43.0 |  |
| Pharmacy | chain of ≤4 pharmacies | 3 | 23.1 | 1 | 4.3 | 25 | 7.0 | 0.002 |
|  | chain of 5-15 pharmacies | 3 | 23.1 | 5 | 21.7 | 67 | 18.8 |  |
|  | chain of ˃15 pharmacies | 5 | 38.5 | 12 | 52.2 | 248 | 69.7 |  |
|  | independently owned | 2 | 15.4 | 5 | 21.7 | 16 | 4.5 |  |
| Pharmacy location | urban area | 11 | 84.6 | 15 | 65.2 | 274 | 77.0 | 0.683 |
|  | suburban area | 1 | 7.7 | 5 | 21.7 | 53 | 14.9 |  |
|  | rural area | 1 | 7.7 | 3 | 13.1 | 29 | 8.1 |  |

Percentages may not add up to 100.0 due to rounding.
