# Supplementary material for: Coping with the burden of the COVID-19 pandemic: a cross-sectional study of community pharmacists from Serbia
Source: BMC Health Serv Res. 2021 Apr 6;21:304. doi: 10.1186/s12913-021-06327-1 (PMC8022120; doi:10.1186/s12913-021-06327-1)
Supplement: Supplementary file 3 — Additional file 3. [file 12913_2021_6327_MOESM3_ESM.docx]

Additional file 3.

Workflow procedures in community pharmacies in Vojvodina during the COVID-19 pandemic by groups.

|  | | Workflow during COVID-19 pandemic | | | | p |
| --- | --- | --- | --- | --- | --- | --- |
|  | | Not changed | | Changed | |  |
|  | | N | % | N | % |  |
| Total | | 27 | 6.9 | 365 | 93.1 |  |
| Gender | male | 5 | 18.5 | 25 | 6.8 | 0.028 |
|  | female | 22 | 81.5 | 340 | 93.2 |  |
| Age, y | <35 | 17 | 63.0 | 190 | 52.1 | 0.538 |
|  | 35-44 | 6 | 22.2 | 111 | 30.4 |  |
|  | 45+ | 4 | 14.8 | 64 | 17.5 |  |
| Experience, y | <10 | 18 | 66.7 | 226 | 61.9 | 0.623 |
|  | 10+ | 9 | 33.3 | 139 | 38.1 |  |
| Job position | responsible pharmacist | 10 | 37.0 | 217 | 59.5 | 0.023 |
|  | pharmacist | 17 | 63.0 | 148 | 40.5 |  |
| Pharmacy | chain of ≤4 pharmacies | 1 | 3.7 | 28 | 7.7 | 0.774 |
|  | chain of 5-15 pharmacies | 4 | 14.8 | 71 | 19.5 |  |
|  | chain of ˃15 pharmacies | 20 | 74.1 | 245 | 67.1 |  |
|  | independently owned | 2 | 7.4 | 21 | 5.8 |  |
| Pharmacy location | urban area | 19 | 70.4 | 281 | 77.0 | 0.732 |
|  | suburban area | 5 | 18.5 | 54 | 14.8 |  |
|  | rural area | 3 | 11.1 | 30 | 8.2 |  |

Percentages may not add up to 100.0 due to rounding.
