# Supplementary material for: Coping with the burden of the COVID-19 pandemic: a cross-sectional study of community pharmacists from Serbia
Source: BMC Health Serv Res. 2021 Apr 6;21:304. doi: 10.1186/s12913-021-06327-1 (PMC8022120; doi:10.1186/s12913-021-06327-1)
Supplement: Supplementary file 4 — Additional file 4. [file 12913_2021_6327_MOESM4_ESM.docx]

Additional file 4.

Interactions with clients in community pharmacies in Vojvodina during the COVID-19 pandemic by groups.

|  | | Interactions with clients during COVID-19 pandemic | | | | | | p |
| --- | --- | --- | --- | --- | --- | --- | --- | --- |
|  | | Less pleasant | | Unchanged | | More pleasant | |  |
|  | | N | % | N | % | N | % |  |
| Total | | 265 | 67.6 | 74 | 18.9 | 53 | 13.5 |  |
| Gender | male | 16 | 6.0 | 7 | 9.5 | 7 | 13.2 | 0.163 |
|  | female | 249 | 94.0 | 67 | 90.5 | 46 | 86.8 |  |
| Age, y | <35 | 159 | 60.0 | 28 | 37.8 | 20 | 37.7 | <0.001 |
|  | 35-44 | 72 | 27.2 | 28 | 37.8 | 17 | 32.1 |  |
|  | 45+ | 34 | 12.8 | 18 | 24.3 | 16 | 30.2 |  |
| Experience, y | <10 | 180 | 67.9 | 40 | 54.1 | 24 | 45.3 | 0.002 |
|  | 10+ | 85 | 32.1 | 34 | 45.9 | 29 | 54.7 |  |
| Job position | responsible pharmacist | 155 | 58.5 | 39 | 52.7 | 33 | 62.3 | 0.529 |
|  | pharmacist | 110 | 41.5 | 35 | 47.3 | 20 | 37.7 |  |
| Pharmacy | chain of ≤4 pharmacies | 16 | 6.0 | 2 | 2.7 | 11 | 20.8 | <0.001 |
|  | chain of 5-15 pharmacies | 45 | 17.0 | 20 | 27.0 | 10 | 18.9 |  |
|  | chain of ˃15 pharmacies | 194 | 73.2 | 45 | 60.8 | 26 | 49.1 |  |
|  | independently owned | 10 | 3.8 | 7 | 9.5 | 6 | 11.3 |  |
| Pharmacy location | urban area | 205 | 77.4 | 52 | 70.3 | 43 | 81.1 | 0.285 |
|  | suburban area | 42 | 15.8 | 12 | 16.2 | 5 | 9.4 |  |
|  | rural area | 18 | 6.8 | 10 | 13.5 | 5 | 9.4 |  |

Percentages may not add up to 100.0 due to rounding.
