# Supplementary material for: Coping with the burden of the COVID-19 pandemic: a cross-sectional study of community pharmacists from Serbia
Source: BMC Health Serv Res. 2021 Apr 6;21:304. doi: 10.1186/s12913-021-06327-1 (PMC8022120; doi:10.1186/s12913-021-06327-1)
Supplement: Supplementary file 5 — Additional file 5. [file 12913_2021_6327_MOESM5_ESM.docx]

Additional file 5.

Availability of protection equipment for community pharmacists in Vojvodina during the COVID-19 pandemic by groups.

|  | | Availability of protection equipment during COVID-19 pandemic | | | | p |
| --- | --- | --- | --- | --- | --- | --- |
|  | | Always available | | Not always available | |  |
|  | | N | % | N | % |  |
| Total | | 188 | 48.0 | 204 | 52.0 |  |
| Gender | male | 14 | 7.4 | 16 | 7.8 | 0.883 |
|  | female | 174 | 92.6 | 188 | 92.2 |  |
| Age, y | <35 | 96 | 51.1 | 111 | 54.4 | 0.035 |
|  | 35-44 | 50 | 26.6 | 67 | 32.8 |  |
|  | 45+ | 42 | 22.3 | 26 | 12.7 |  |
| Experience, y | <10 | 111 | 59.0 | 133 | 65.2 | 0.209 |
|  | 10+ | 77 | 41.0 | 71 | 34.8 |  |
| Job position | responsible pharmacist | 110 | 58.5 | 117 | 57.4 | 0.817 |
|  | pharmacist | 78 | 41.5 | 87 | 42.6 |  |
| Pharmacy | chain of ≤4 pharmacies | 14 | 7.4 | 15 | 7.4 | 0.888 |
|  | chain of 5-15 pharmacies | 39 | 20.7 | 36 | 17.6 |  |
|  | chain of ˃15 pharmacies | 124 | 66.0 | 141 | 69.1 |  |
|  | independently owned | 11 | 5.9 | 12 | 5.9 |  |
| Pharmacy location | urban area | 147 | 78.2 | 153 | 75.0 | 0.647 |
|  | suburban area | 25 | 13.3 | 34 | 16.7 |  |
|  | rural area | 16 | 8.5 | 17 | 8.3 |  |

Percentages may not add up to 100.0 due to rounding.
